# A prediction model for in-hospital mortality in intensive care unit patients with metastatic cancer

**DOI:** 10.3389/fsurg.2023.992936

**Published:** 2023-01-30

**Authors:** Meizhen Wu, Haijin Gao

**Affiliations:** ^1^ Department of Intensive Care Unit, Shanxi Province Cancer Hospital/Shanxi Hospital Affiliated to Cancer Hospital, Chinese Academy of Medical Sciences/Cancer Hospital Affiliated to Shanxi Medical University, Taiyuan, China; ^2^ Department of Intensive Care Unit, The First Hospital of Shanxi Medical University, Taiyuan, China

**Keywords:** nomogram, in-hospital mortality, intensive care unit, metastatic cancer, MIMIC-III

## Abstract

**Aim:**

To identify predictors for in-hospital mortality in patients with metastatic cancer in intensive care units (ICUs) and established a prediction model for in-hospital mortality in those patients.

**Methods:**

In this cohort study, the data of 2,462 patients with metastatic cancer in ICUs were extracted from the Medical Information Mart for Intensive Care III (MIMIC-III) database. Least absolute shrinkage and selection operator (LASSO) regression analysis was applied to identify the predictors for in-hospital mortality in metastatic cancer patients. Participants were randomly divided into the training set (*n* = 1,723) and the testing set (*n* = 739). Patients with metastatic cancer in ICUs from MIMIC-IV were used as the validation set (*n* = 1,726). The prediction model was constructed in the training set. The area under the curve (AUC), sensitivity, specificity, positive predictive value (PPV), and negative predictive value (NPV) were employed for measuring the predictive performance of the model. The predictive performance of the model was validated in the testing set and external validation was performed in the validation set.

**Results:**

In total, 656 (26.65%) metastatic cancer patients were dead in hospital. Age, respiratory failure, the sequential organ failure assessment (SOFA) score, the Simplified Acute Physiology Score II (SAPS II) score, glucose, red cell distribution width (RDW) and lactate were predictors for the in-hospital mortality in patients with metastatic cancer in ICUs. The equation of the prediction model was ln(*P*/(1 + *P*)) = −5.9830 + 0.0174 × age + 1.3686 × respiratory failure + 0.0537 × SAPS II + 0.0312 × SOFA + 0.1278 × lactate − 0.0026 × glucose + 0.0772 × RDW. The AUCs of the prediction model was 0.797 (95% CI,0.776–0.825) in the training set, 0.778 (95% CI, 0.740–0.817) in the testing set and 0.811 (95% CI, 0.789–0.833) in the validation set. The predictive values of the model in lymphoma, myeloma, brain/spinal cord, lung, liver, peritoneum/pleura, enteroncus and other cancer populations were also assessed.

**Conclusion:**

The prediction model for in-hospital mortality in ICU patients with metastatic cancer exhibited good predictive ability, which might help identify patients with high risk of in-hospital death and provide timely interventions to those patients.

## Introduction

The number of cancer patients has steadily been increasing and cancer has become the major cause of death in many countries (1). The American Cancer Society estimated that 1,898,160 new cancer cases and 608,570 cancer deaths are projected to occur in the United States in 2021 (2). According to the data from the World Health Organization (WHO), the global incidence of cancer might increase by over 63% in 2040 relative to 2018 (3). Approximately 5%–10% of cancer patients may develop a life-threatening disease which requires intensive care unit (ICU) admission (4). A previous study indicated that nearly 1/7 of the patients in ICUs had cancers in Europe (5). Cancer patients admitted into ICUs were associated with poor outcomes with a hospital mortality as high as 50% (6). In recent years, more and more patients with metastatic cancer actively received the treatments and many of them may be admitted to ICUs during the course of their disease, and the prognosis of these patients was even poorer (7). To accurately identify patients with metastatic cancer who are at high risk of mortality was essential for clinicians to provide timely interventions and improve the outcomes of these patients.

Previously, several studies have identified various predictors for the mortality in patients with metastatic cancers (8–10). Additionally, Lee Cheng et al. established a prediction model for predicting the 14-days' mortality in patients with metastatic or advanced cancer based on the laboratory test results of these patients at hospital admission in ICUs (11). This study included patients with metastatic or advanced cancer, but the characteristics of patients with metastatic or advanced cancers were not the same (12). Cancer metastasis includes both regional lymph node metastasis, non-regional lymph node metastasis, and distant metastasis. Patients with cancers at advanced stage are metastatic, which generally refers to distant metastasis (12, 13). Additionally, the discriminatory ability of the model was moderate. A suitable model for predicting the mortality of ICU patients with metastatic cancer was still required.

In the current study, we planned to establish a prediction model for in-hospital mortality in those patients based on the data from the Medical Information Mart for Intensive Care III (MIMIC-III). The predictive value of the model for different cancers were also evaluated. Internal validation of the performance of the model was assessed. External validation of the performance of the model was conducted using the data from MIMIC-IV. The findings of this study might provide a tool for identify patients with high risk of in-hospital death in ICU patients with metastatic cancer.

## Materials and methods

### Study design and population

In this cohort study, the data of 2,462 patients ≥18 years with metastatic cancer admitted into ICUs were extracted from MIMIC-III database and the data of 1,726 patients ≥18 years with metastatic cancer admitted into ICUs were extracted from MIMIC-IV database. MIMIC-III is a freely accessed database including the demographic characteristics, vital signs, laboratory test results, imaging examinations, and a data dictionary if patients admitted to the Beth Israel Deaconess Medical Center in Boston, Massachusetts (14). A complete hospitalization record of each patient can be obtained by using a special code assigned in each hospital at ICU admission, and hospital staff entered the final precise diagnosis based on the International Classification of Disease 9th Edition (ICD-9) when patients were discharged. MIMIC-IV is an updated version of MIMIC-III that simplified the structure, added new data elements, and improved the usability of previous data elements (15). This study was conducted using an anonymized public database that satisfied review committee agreements, and the requirement for ethical consent was not necessary.

### Potential predictors

The potential predictors for the in-hospital mortality in patients with metastatic cancer admitted into ICUs included baseline characteristics, treatment associated characteristics and clinical characteristics. Some baseline and clinical variables analyzed as potential predictors used the first measurement data measured within 24 h ICU admission.

Baseline characteristics included age (years), gender, race (White, Black, Asian, Hispanic or others), marital status (married, single, widowed or divorced/separated), history of comorbidities included congestive heart failure, cardiac dysrhythmia, atrial fibrillation, chronic kidney disease (CKD), myocardial infarction, diabetes mellitus, hypertension, hyperlipidemia, and septicemia, tumor type (lymphoa, myeloma, brain/spinal cord, lung, liver, peritoneum/pleura, enteroncus or others) and respiratory failure.

Treatment associated characteristics included surgery, chemotherapy, radiotherapy, ventilation, partial arterial oxygen pressure (PaO_2_)/the fraction of inspired oxygen (FiO_2_), the Simplified Acute Physiology Score II (SAPS II) score, the Glasgow Coma Scale (GCS) score, the sequential organ failure assessment (SOFA) score.

Clinical characteristics included temperature (°C), heart rate (time/minute), systolic blood pressure (SBP, mmHg), diastolic blood pressure (DBP, mmHg), mean arterial pressure (MAP, mmHg), red blood count (RBC, m/µl), sodium (mEq/L), potassium (mEq/L), phosphate (mg/dl), calcium (mg/dl), platelets (PLT, K/µl), pondus hydrogenii (pH), lactate (mmol/L), international normalized ratio (INR), albumin (g/L), hemoglobin (g/dL), white blood cell count (WBC, K/µL), glucose (mg/dl), creatinine (mg/dl), blood urea nitrogen (BUN, mg/dl), bicarbonate (mEq/l), neutrophil, lymphocytes, total bilirubin (TBIL, μmol/L), hematocrit (HCT, L/L), and red cell distribution width (RDW).

### Outcome variables

The outcome in our study was patients with metastatic cancer died in hospital or discharged from hospital. The medium follow-up time was 6.63 (3.78, 10.67) days. The follow-up was terminated when the outcome occurred.

### Data extraction

The data of 2,462 patients with metastatic cancer admitted into ICUs were extracted from the MIMIC-III v1.3. The numbers of patients and ICD-9 code of different cancers were shown in Supplementary Table S1. The missing values were exhibited in Supplementary Table S2. Sensitivity analysis was conducted and the results depicted that no statistical difference was observed between the data before and after manipulation (Supplementary Table S2).

### Construction of the prediction model

Participants from MIMIC-III were randomly divided into the training set (*n* = 1,723) and the testing set (*n* = 739). Least absolute shrinkage and selection operator (LASSO) regression analysis was used for identifying the factors with statistical difference between the survival group and the death group in the training set (*P *< 0.05) to screen the predictors of mortality in ICU patients with metastatic cancer. The predictors were included in the prediction model and area under the curve (AUC), sensitivity, specificity, positive predictive value (PPV), and negative predictive value (NPV) were employed for measuring the predictive performance of the model. ROC curve and Calibration curve were plotted to evaluate the predictive value of the model. Subgroup analysis was conducted in terms of different cancer types. The data in the testing set were used as internal validation of the performance of the model and the data from MIMIC-IV (*n* = 1,726) were applied as external validation of the value of the model.

### Statistical analysis

The Shapiro test was applied to test the normality of measurement data. The measurement data with normal distribution were described as Mean ± standard deviation (SD) and t test was applied for comparisons between groups. Non-normal distributed data were shown as median and quartile spacing *M* (*Q*_1_, *Q*_3_), and comparisons between groups was performed by Mann-Whitney U rank-sum test. The enumeration data were displayed as *n* (%), and the differences between groups were conducted *via χ*^2^ test or Fisher's exact probability method. The missing values were manipulated *via* multiple imputation (R: mice) and differences of variables before and after interpolation were analyzed by sensitivity analysis (Supplementary Table S2). The data from MIMIC-III were randomly divided into the training set and the testing set at 7:3 *via* SAS 9.4 generated random number. All statistical tests were performed by a two-sided test. SAS 9.4 was used to screen the predictors and construct the prediction model. The validation and nomogram of the model were performed by R 4.0.2. *P *< 0.05 was set as statistical difference.

## Results

### Equilibrium test in the training set and the testing set

**Figure 1:**
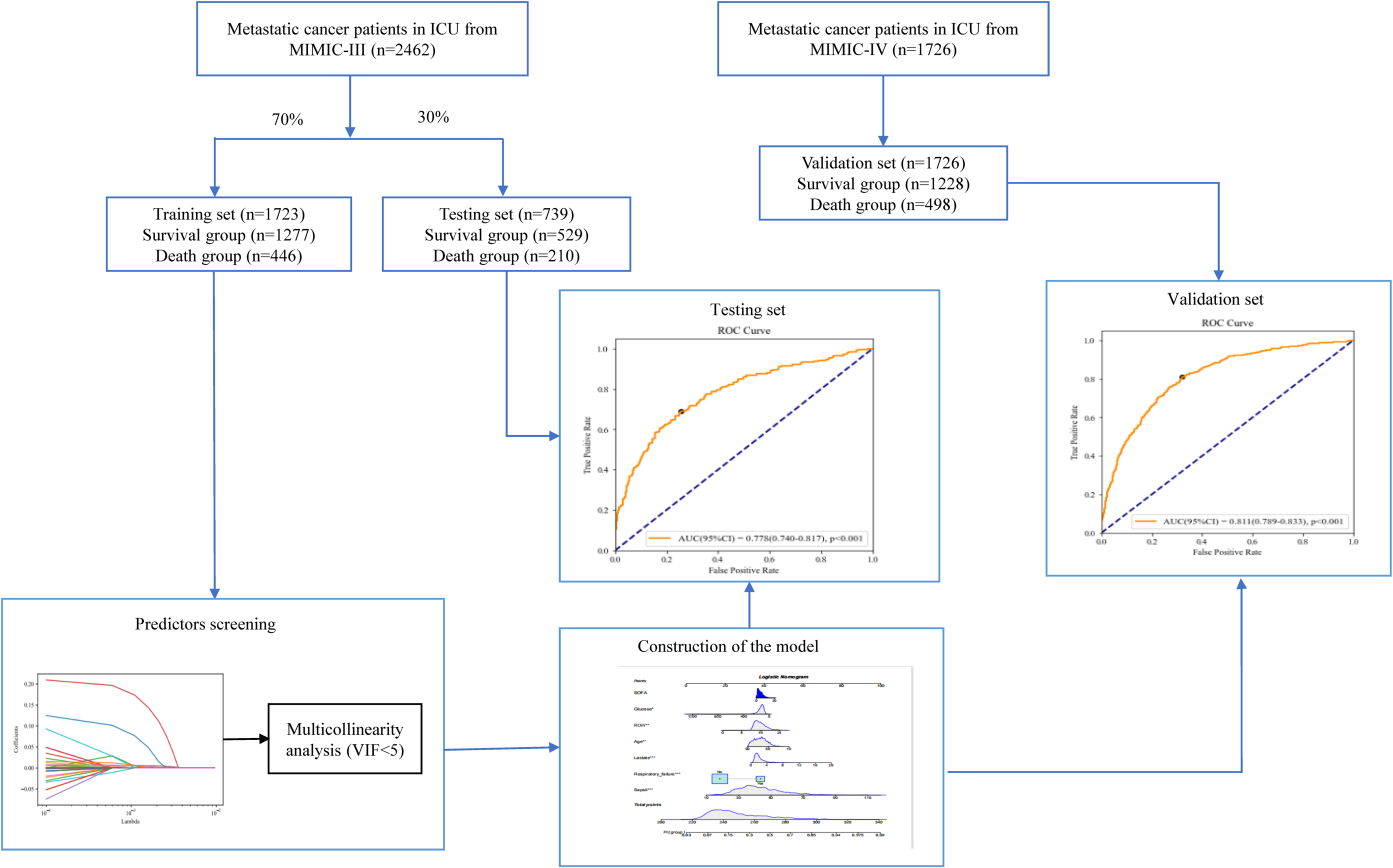
The screen process of participants.

In total, 2,530 patients with metastatic cancer admitted into ICUs from MIMIC-III were included in this study. Patients <18 years were excluded (*n* = 68) and 2,462 patients finally participated in. The screen process was displayed in Figure 1. The average age of all subjects were 65.28 ± 12.65 years, including 1,312 males (53.29%) and 1,150 females (46.71%). Among all patients, 1996 patients were White, accounting for 81.07%, 201 subjects were Black, accounting for 8.16%, 114 persons were Asia, accounting for 4.63%, 58 people were the Spanish, accounting for 2.36%, and 93 participants were other races, accounting for 3.78%. 1504 participants were married (61.09%), 500 subjects were single (20.31%), 290 people were widowed (11.78%), and 168 patients were divorced/separated (6.82%). 382 patients were complicated with congestive heart failure, accounting for 15.52%, 143 persons had arrhythmias, accounting for 5.81%, 584 subjects had respiratory failure, accounting for 23.72%, and 455 participants were complicated with sepsis, accounting for 18.48%. In terms of cancer types, 323 patients were lymphoma (13.12%), 325 patients were myeloma (13.20%), 376 people were brain/spinal cancer (15.27%), 273 persons were lung cancer (11.09%), 328 patients were liver cancer (13.32%), 245 subjects were peritoneum/pleura cancer (9.95%), 103 participants were enteroncus (4.18%), and 489 patients were others (19.86%). In all participants, 656 people died, accounting for 26.65%, and 1806 were survived, accounting for 73.35%. The equilibrium test revealed that there was no significant difference in the baseline, treatment and clinical characteristics between the training set and the testing set (All *P *> 0.05) (Table 1).

**Table 1:** The equilibrium test in the training set and the testing set.

Characteristic	Total (*n* = 2462)	Group		Statistical magnitude	*P*
		Training set (*n* = 1723)	Testing set (*n* = 739)		
Baseline characteristics					
Age, Mean ± SD	65.28 ± 12.65	65.19 ± 12.66	65.49 ± 12.64	*t* = −0.540	0.591
Gender, *n* (%)				*χ*^2 ^= 0.656	0.418
Female	1,150 (46.71)	814 (47.24)	336 (45.47)		
Male	1,312 (53.29)	909 (52.76)	403 (54.53)		
Race, *n* (%)				χ^2 ^= 8.904	0.064
White	1,996 (81.07)	1,403 (81.43)	593 (80.24)		
Black	201 (8.16)	135 (7.84)	66 (8.93)		
Asian	114 (4.63)	82 (4.76)	32 (4.33)		
Hispanic	58 (2.36)	32 (1.86)	26 (3.52)		
Others	93 (3.78)	71 (4.12)	22 (2.98)		
Marital, *n* (%)				χ^2 ^= 1.684	0.640
Married	1,504 (61.09)	1,050 (60.94)	454 (61.43)		
Single	500 (20.31)	349 (20.26)	151 (20.43)		
Widowed	290 (11.78)	211 (12.25)	79 (10.69)		
Divorced/separated	168 (6.82)	113 (6.56)	55 (7.44)		
Insurance, *n* (%)				χ^2 ^= 4.129	0.127
Medicare	1,280 (51.99)	875 (50.78)	405 (54.80)		
Private	272 (11.05)	201 (11.67)	71 (9.61)		
Others	910 (36.96)	647 (37.55)	263 (35.59)		
Congestive heart failure, *n* (%)	382 (15.52)	265 (15.38)	117 (15.83)	χ^2 ^= 0.081	0.776
Cardiac dysrhythmia, *n* (%)	143 (5.81)	99 (5.75)	44 (5.95)	χ^2 ^= 0.041	0.840
Respiratory failure, *n* (%)	584 (23.72)	388 (22.52)	196 (26.52)	χ^2 ^= 3.581	0.052
Atrial fibrillation, *n* (%)	485 (19.70)	328 (19.04)	157 (21.24)	χ^2 ^= 1.594	0.207
CKD, *n* (%)	226 (9.18)	161 (9.34)	65 (8.80)	χ^2 ^= 0.187	0.666
Myocardial infarction, *n* (%)	128 (5.20)	81 (4.70)	47 (6.36)	χ^2 ^= 2.887	0.089
Diabetes mellitus, *n* (%)	412 (16.73)	302 (17.53)	110 (14.88)	χ^2 ^= 2.592	0.107
Hypertension, *n* (%)	1,025 (41.63)	729 (42.31)	296 (40.05)	χ^2 ^= 1.083	0.298
Hyperlipidemia, *n* (%)	637 (25.87)	449 (26.06)	188 (25.44)	χ^2 ^= 0.103	0.748
Septicemia, *n* (%)	455 (18.48)	325 (18.86)	130 (17.59)	χ^2 ^= 0.555	0.456
First care unit, *n* (%)				χ^2 ^= 2.912	0.573
CCU	167 (6.78)	118 (6.85)	49 (6.63)		
CSRU	96 (3.90)	71 (4.12)	25 (3.38)		
MICU	1,354 (55.00)	939 (54.50)	415 (56.16)		
SICU	589 (23.92)	423 (24.55)	166 (22.46)		
TSICU	256 (10.40)	172 (9.98)	84 (11.37)		
Tumor type, *n* (%)				χ^2 ^= 2.405	0.934
Lymphoma	323 (13.12)	226 (13.12)	97 (13.13)		
Myeloma	325 (13.20)	232 (13.46)	93 (12.58)		
Brain/spinal cord	376 (15.27)	271 (15.73)	105 (14.21)		
Lung	273 (11.09)	191 (11.09)	82 (11.10)		
Liver	328 (13.32)	226 (13.12)	102 (13.80)		
Peritoneum/pleura	245 (9.95)	164 (9.52)	81 (10.96)		
Enteroncus	103 (4.18)	71 (4.12)	32 (4.33)		
Other	489 (19.86)	342 (19.85)	147 (19.89)		
Treatment characteristics					
Surgery, *n* (%)	1,980 (80.42)	1,394 (80.91)	586 (79.30)	χ^2 ^= 0.850	0.356
Chemotherapy, *n* (%)	98 (3.98)	68 (3.95)	30 (4.06)	χ^2 ^= 0.017	0.895
Radiotherapy, *n* (%)	79 (3.21)	55 (3.19)	24 (3.25)	χ^2 ^= 0.005	0.943
Length of hospital, *M* (*Q*_1_, *Q*_3_)	7.89 (4.23,12.63)	7.77 (4.23,12.60)	8.01 (4.23,12.81)	Z = 0.433	0.665
Length of ICU, *M* (*Q*_1_, *Q*_3_)	2.07 (1.19,4.01)	2.07 (1.17,3.92)	2.11 (1.25,4.18)	Z = 0.930	0.353
Ventilation, *n* (%)	642 (26.08)	436 (25.30)	206 (27.88)	χ^2 ^= 1.773	0.183
PaO_2_/FiO_2_, *M* (*Q*_1_, *Q*_3_)	333.33 (234.00,422.50)	334.00 (232.00,424.00)	326.00 (235.00,420.00)	*Z* = −0.558	0.577
SAPS II score, Mean ± SD	43.65 ± 14.12	43.52 ± 14.17	43.96 ± 14.02	*t* = −0.710	0.477
GCS score, *M* (*Q*_1_, *Q*_3_)	12.15 ± 3.83	12.20 ± 3.80	12.06 ± 3.90	t = 0.820	0.413
SOFA score, *M* (*Q*_1_, *Q*_3_)	4.00 (2.00,6.00)	4.00 (2.00,6.00)	4.00 (2.00,6.00)	Z = 1.431	0.152
Clinical characteristics					
Temperature, Mean ± SD	36.73 ± 1.90	36.76 ± 2.20	36.65 ± 0.83	t = 1.680	0.094
Heart Rate, Mean ± SD	96.13 ± 20.32	96.37 ± 20.23	95.56 ± 20.52	t = 0.910	0.361
SBP, Mean ± SD	125.16 ± 22.34	125.10 ± 22.56	125.30 ± 21.82	*t* = −0.210	0.833
DBP, Mean ± SD	66.03 ± 14.90	66.00 ± 14.80	66.10 ± 15.13	*t* = −0.160	0.875
MAP, Mean ± SD	82.53 ± 17.86	82.48 ± 17.62	82.66 ± 18.44	*t* = −0.230	0.817
RBC, Mean ± SD	3.74 ± 0.75	3.76 ± 0.75	3.70 ± 0.76	*t* = 1.640	0.102
Sodium, Mean ± SD	136.70 ± 5.25	136.72 ± 5.09	136.66 ± 5.60	*t* = 0.250	0.806
Potassium, Mean ± SD	4.35 ± 0.85	4.37 ± 0.86	4.32 ± 0.82	*t* = 1.330	0.182
Phosphate, *M* (*Q*_1_, *Q*_3_)	3.50 (2.90,4.20)	3.50 (2.90,4.20)	3.60 (3.00,4.20)	*Z* = 0.793	0.428
Calcium, Mean ± SD	8.61 ± 1.03	8.61 ± 1.02	8.62 ± 1.06	*t* = −0.110	0.915
PLT, *M* (*Q*_1_, *Q*_3_)	259.00 (179.00,363.00)	259.00 (179.00,361.00)	261.00 (180.00,366.00)	*Z* = 0.293	0.770
pH, Mean ± SD	7.39 ± 0.09	7.39 ± 0.09	7.39 ± 0.09	*t* = −0.610	0.543
Lactate, *M* (*Q*_1_, *Q*_3_)	1.70 (1.30,2.60)	1.70 (1.30,2.70)	1.70 (1.30,2.50)	*Z* = −0.694	0.488
INR, *M* (*Q*_1_, *Q*_3_)	1.20 (1.10,1.40)	1.20 (1.10,1.40)	1.20 (1.10,1.40)	Z = 0.460	0.645
Albumin, Mean ± SD	3.05 ± 0.67	3.07 ± 0.68	3.00 ± 0.67	t = 1.600	0.114
Hemoglobin, Mean ± SD	11.05 ± 2.20	11.10 ± 2.18	10.95 ± 2.24	t = 1.570	0.116
WBC, *M* (*Q*_1_, *Q*_3_)	10.20 (7.10,14.70)	10.40 (7.20,14.70)	9.80 (6.70,14.60)	*Z* = −1.465	0.143
Glucose, *M* (*Q*_1_, *Q*_3_)	126.00 (104.00,157.00)	125.00 (105.00,157.00)	127.00 (104.00,157.00)	*Z* = −0.002	0.998
Creatinine, *M* (*Q*_1_, *Q*_3_)	0.90 (0.70,1.30)	0.90 (0.70,1.30)	0.90 (0.70,1.30)	Z = 0.133	0.894
BUN, *M* (*Q*_1_, *Q*_3_)	20.00 (14.00,30.00)	19.00 (14.00,29.00)	20.00 (14.00,31.00)	Z = 0.448	0.654
Bicarbonate, Mean ± SD	24.18 ± 4.70	24.07 ± 4.78	24.45 ± 4.48	*t* = −1.870	0.061
Neutrophil, Mean ± SD	79.39 ± 14.63	79.58 ± 14.25	78.97 ± 15.48	*t* = 0.920	0.358
Lymphocytes, *M* (*Q*_1_, *Q*_3_)	9.00 (5.00,15.60)	9.00 (5.00,15.40)	9.20 (5.10,15.70)	*Z* = 0.727	0.468
TBIL, *M* (*Q*_1_, *Q*_3_)	0.60 (0.40,1.10)	0.60 (0.40,1.10)	0.60 (0.40,1.10)	*Z* = 0.297	0.767
Hematocrit, Mean ± SD	33.15 ± 6.22	33.29 ± 6.16	32.84 ± 6.37	*t* = 1.640	0.100
RDW, Mean ± SD	15.96 ± 2.55	15.95 ± 2.54	16.00 ± 2.57	*t* = −0.480	0.629
Group, *n* (%)				χ^2 ^= 1.696	0.193
Alive	1,806 (73.35)	1,277 (74.11)	529 (71.58)		
Dead	656 (26.65)	446 (25.89)	210 (28.42)		

CKD, chronic kidney disease; CCU, critical care unit; CSRU, Cardiac Surgery Recovery Unit; MICU, medical intensive care unit; SICU, surgical intensive care unit; TSICU, trauma or surgical intensive care unit; PaO_2_/FiO_2_, partial arterial oxygen pressure/the fraction of inspired oxygen; SAPS II, the Simplified Acute Physiology Score II; GCS, Glasgow Coma Scale; SOFA, sequential organ failure assessment; SBP, systolic blood pressure; MAP, mean arterial pressure; RBC, red blood count; PLT, platelets; INR, international normalized ratio; WBC, white blood cell count; BUN, blood urea nitrogen; TBIL, total bilirubin; RDW, red cell distribution width.

Non-normal distributed data were shown as *M* (*Q*_1_, *Q*_3_).

### Comparisons of the characteristics in patients between the survival group and the death group in the training set

The proportions of patients with respiratory failure in death group (47.53% vs. 13.76%, *χ*^2 ^= 215.814, *P *< 0.001), patients with myocardial infarction (6.95% vs. 3.92%, *χ*^2 ^= 6.797, *P *= 0.009), patients with septicemia (37.89% vs. 12.22%, *χ*^2 ^= 142.391, *P *< 0.001), patients with chemotherapy (5.83% vs. 3.29%, *χ*^2 ^= 5.628, *P *= 0.018), and mechanical ventilation (31.61% vs. 23.10%, *χ*^2 ^= 12.675, *P *< 0.001) were higher than in the survival group. The median LOS in ICU (2.63 day vs. 2.00 day, *Z* = 5.060, *P *< 0.001), SAPS II score (52.87 vs. 40.26, *t* = −15.140, *P *< 0.001), SOFA scores (6.00 vs. 3.00, *Z* = 13.362, *P *< 0.001), heart rate (102.75 times/minute vs. 94.15 times/minute, *t* = −7.480, *P *< 0.001), potassium (4.58 mEq/l vs. 4.29 mEq/l, *t* = 5.460, *P *< 0.001), phosphate (3.80 mg/dl vs. 3.50 mg/dl, *Z* = 5.334, *P *< 0.001) and lactate (2.20 mmol/l vs. 1.70 mmol/l, *Z* = 9.100, *P *< 0.001), INR (1.30 vs. 1.20, *Z* = 7.692, *P *< 0.001), WBC (11.5 K/uL vs. 9.9 K/uL, *Z* = 4.582, *P *< 0.001), creatinine (1.00 mg/dl vs. 0.90 mg/dl, *Z* = 5.893, *P *< 0.001), BUN (26.00 mg/dl vs. 18.00 mg/dl, *Z* = 10.677, *P *< 0.001), TBIL (0.61 *μ*mol/l vs. 0.60 *μ*mol/l, Z = 2.843, *P *= 0.004) and RDW(16.60 vs. 15.72, *t* = −6.090, *P* < 0.001) in the death group were higher than in the survival group. The proportion of patients with hyperlipidemia (19.06% vs. 28.50%, *χ*^2 ^= 15.307, *P *< 0.001) in the death group was lower than in the survival group. The median LOS in hospital (6.05 days vs. 8.21 days, *Z* = −7.474, *P *< 0.001), PaO_2_/FiO_2_ (285.00 vs. 351.43, *Z* = 7.418, *P *< 0.001), GCS score (13.99 vs. 14.00, *Z* = 7.632, *P *< 0.001), SBP (119.05 mmHg vs. 127.21 mmHg, *t* = 7.070, *P *< 0.001), MAP (78.54 mmHg vs. 83.85 mmHg, *t* = 5.530, *P *< 0.001), RBC (3.70 m/µl vs. 3.78 m/µl, *t* = 2.030, *P* = 0.043), sodium (135.59 mEq/L vs. 137.12 mEq/L, *t* = 4.900, *P *< 0.001), calcium (8.51 mg/dl vs. 8.65 mg/dl, *t* = 2.170, *P *= 0.030), PLT (249.50 K/µl vs. 262.00 K/µl, *Z* = 2.042, *P *= 0.040), pH (7.36 vs. 7.40, *t* = 7.460, *P *< 0.001), albumin (1.30 g/L vs. 1.20 g/L, *T* = 7.460, *P *< 0.001), hemoglobin (10.85 g/dl vs. 11.18 g/dl, *t* = 2.800, *P *= 0.005), glucose (120.00 mg/dl vs. 128.00 mg/dl, *Z* = −2.919, *P *= 0.004), bicarbonate (23.15 mEq/L vs. 24.39 mEq/L, *t* = 4.270, *P* < 0.001), and lymphocytes (6.80 vs. 10.00, *Z* = 8.188, *P *< 0.001) in the death group were lower than the survival group. The race (*χ*^2 ^= 15.991, *P *= 0.003), first care unit (*χ*^2 ^= 87.775, *P *< 0.001) and tumor type (*χ*^2 ^= 53.144, *P *< 0.001) were statistically different between the death group and the survival group (Table 2).

**Table 2:** Comparisons of the characteristics in patients between the survival group and the death group in the training set.

Characteristic	Total (*n* = 1723)	Prognosis		Statistical magnitude	*P*
		Survival group (*n* = 1277)	Death group (*n* = 446)		
Baseline characteristics					
Age, Mean ± SD	65.19 ± 12.66	65.03 ± 12.40	65.64 ± 13.36	*t* = −0.880	0.380
Gender, *n* (%)				χ^2 ^= 0.920	0.338
Female	814 (47.24)	612 (47.92)	202 (45.29)		
Male	909 (52.76)	665 (52.08)	244 (54.71)		
Race, *n* (%)				χ^2 ^= 15.991	0.003
White	1,403 (81.43)	1,063 (83.24)	340 (76.23)		
Black	135 (7.84)	86 (6.73)	49 (10.99)		
Asian	82 (4.76)	51 (3.99)	31 (6.95)		
Hispanic	32 (1.86)	25 (1.96)	7 (1.57)		
Others	71 (4.12)	52 (4.07)	19 (4.26)		
Marital, *n* (%)				χ^2 ^= 1.347	0.718
Married	1,050 (60.94)	771 (60.38)	279 (62.56)		
Single	349 (20.26)	267 (20.91)	82 (18.39)		
Widowed	211 (12.25)	155 (12.14)	56 (12.56)		
Divorced/separated	113 (6.56)	84 (6.58)	29 (6.50)		
Congestive heart failure, *n* (%)	265 (15.38)	187 (14.64)	78 (17.49)	χ^2 ^= 2.056	0.152
Cardiac dysrhythmia, *n* (%)	99 (5.75)	76 (5.95)	23 (5.16)	χ^2 ^= 0.385	0.535
Respiratory failure, *n* (%)	388 (22.52)	176 (13.78)	212 (47.53)	χ^2 ^= 215.814	<0.001
Atrial fibrillation, *n* (%)	328 (19.04)	230 (18.01)	98 (21.97)	χ^2 ^= 3.367	0.067
CKD, *n* (%)	161 (9.34)	114 (8.93)	47 (10.54)	χ^2 ^= 1.013	0.314
Myocardial infarction, *n* (%)	81 (4.70)	50 (3.92)	31 (6.95)	χ^2 ^= 6.797	0.009
Diabetes mellitus, *n* (%)	302 (17.53)	230 (18.01)	72 (16.14)	χ^2 ^= 0.797	0.372
Hypertension, *n* (%)	729 (42.31)	558 (43.70)	171 (38.34)	χ^2 ^= 3.884	0.049
Hyperlipidemia, *n* (%)	449 (26.06)	364 (28.50)	85 (19.06)	χ^2 ^= 15.307	<0.001
Septicemia, *n* (%)	325 (18.86)	156 (12.22)	169 (37.89)	χ^2 ^= 142.391	<0.001
Tumor type, *n* (%)				χ^2 ^= 53.144	<0.001
Lymphoma	226 (13.12)	192 (15.04)	34 (7.62)		
Myeloma	232 (13.46)	173 (13.55)	59 (13.23)		
Brain/spinal cord	271 (15.73)	228 (17.85)	43 (9.64)		
Lung	191 (11.09)	127 (9.95)	64 (14.35)		
Liver	226 (13.12)	154 (12.06)	72 (16.14)		
Peritoneum/pleura	164 (9.52)	101 (7.91)	63 (14.13)		
Enteroncus	71 (4.12)	56 (4.39)	15 (3.36)		
Other	342 (19.85)	246 (19.26)	96 (21.52)		
Treatment characteristics					
Surgery, *n* (%)	1,394 (80.91)	1,044 (81.75)	350 (78.48)	χ^2 ^= 2.300	0.129
Chemotherapy, *n* (%)	68 (3.95)	42 (3.29)	26 (5.83)	χ^2 ^= 5.628	0.018
Radiotherapy, *n* (%)	55 (3.19)	39 (3.05)	16 (3.59)	χ^2 ^= 0.304	0.581
Length of hospital, *M* (*Q*_1_, *Q*_3_)	7.77 (4.23,12.60)	8.21 (5.03,12.99)	6.05 (2.13,10.89)	*Z* = −7.474	<0.001
Length of ICU, *M* (*Q*_1_, *Q*_3_)	2.07 (1.17,3.92)	2.00 (1.14,3.37)	2.63 (1.41,5.36)	*Z* = 5.060	<0.001
Ventilation, *n* (%)	436 (25.30)	295 (23.10)	141 (31.61)	χ^2 ^= 12.675	<0.001
PaO_2_/FiO_2_, *M* (*Q*_1_, *Q*_3_)	334.00 (232.00,424.00)	351.43 (249.00,438.00)	285.00 (174.00,384.00)	*Z* = −7.418	<0.001
SAPS II score, Mean ± SD	43.52 ± 14.17	40.26 ± 11.78	52.87 ± 16.15	*t* = −15.140	<0.001
GCS score, *M* (*Q*_1_, *Q*_3_)	14.00 (10.00,15.00)	14.00 (11.00,15.00)	13.00 (7.00,15.00)	*Z* = −7.632	<0.001
SOFA score, *M* (*Q*_1_, *Q*_3_)	4.00 (2.00,6.00)	3.00 (2.00,5.00)	6.00 (3.00,9.00)	*Z* = 13.362	<0.001
Clinical characteristics					
Temperature, Mean ± SD	36.76 ± 2.20	36.80 ± 2.50	36.65 ± 0.86	*t* = 1.860	0.063
Heart Rate, Mean ± SD	96.37 ± 20.23	94.15 ± 19.32	102.75 ± 21.42	*t* = −7.480	<0.001
SBP, Mean ± SD	125.10 ± 22.56	127.21 ± 22.96	119.05 ± 20.22	*t* = 7.070	<0.001
DBP, Mean ± SD	66.00 ± 14.80	66.36 ± 14.46	64.94 ± 15.69	*t* = 1.680	0.093
MAP, Mean ± SD	82.48 ± 17.62	83.85 ± 17.60	78.54 ± 17.09	*t* = 5.530	<0.001
RBC, Mean ± SD	3.76 ± 0.75	3.78 ± 0.74	3.70 ± 0.78	*t* = 2.030	0.043
Sodium, Mean ± SD	136.72 ± 5.09	137.12 ± 4.70	135.59 ± 5.96	*t* = 4.900	<0.001
Potassium, Mean ± SD	4.37 ± 0.86	4.29 ± 0.80	4.58 ± 0.99	*t* = −5.460	<0.001
Phosphate, *M* (*Q*_1_, *Q*_3_)	3.50 (2.90,4.20)	3.50 (2.90,4.10)	3.80 (3.00,4.90)	*Z* = 5.334	<0.001
Calcium, Mean ± SD	8.61 ± 1.02	8.65 ± 0.96	8.51 ± 1.15	*t* = 2.170	0.030
PLT, *M* (*Q*_1_, *Q*_3_)	259.00 (179.00,361.00)	262.00 (189.00,356.00)	249.50 (157.00,371.00)	*Z* = −2.042	0.041
pH, Mean ± SD	7.39 ± 0.09	7.40 ± 0.08	7.36 ± 0.11	*t* = 7.460	<0.001
Lactate, *M* (*Q*_1_, *Q*_3_)	1.70 (1.30,2.70)	1.70 (1.20,2.40)	2.20 (1.40,3.70)	*Z* = 9.100	<0.001
INR, *M* (*Q*_1_, *Q*_3_)	1.20 (1.10,1.40)	1.20 (1.10,1.40)	1.30 (1.10,1.60)	*Z* = 7.692	<0.001
Albumin, Mean ± SD	3.07 ± 0.68	3.16 ± 0.67	2.81 ± 0.64	*t* = 9.600	<0.001
Hemoglobin, Mean ± SD	11.10 ± 2.18	11.18 ± 2.17	10.85 ± 2.20	*t* = 2.800	0.005
WBC, *M* (*Q*_1_, *Q*_3_)	10.40 (7.20,14.70)	9.90 (7.10,13.90)	11.50 (7.60,16.90)	*Z* = 4.582	<0.001
Glucose, *M* (*Q*_1_, *Q*_3_)	125.00 (105.00,157.00)	128.00 (107.00,158.00)	120.00 (100.00,155.00)	*Z* = −2.919	0.004
Creatinine, *M* (*Q*_1_, *Q*_3_)	0.90 (0.70,1.30)	0.90 (0.70,1.20)	1.00 (0.70,1.70)	*Z* = 5.893	<0.001
BUN, *M* (*Q*_1_, *Q*_3_)	19.00 (14.00,29.00)	18.00 (13.00,25.00)	26.00 (17.00,43.00)	*Z* = 10.677	<0.001
Bicarbonate, Mean ± SD	24.07 ± 4.78	24.39 ± 4.43	23.15 ± 5.57	*t* = 4.270	<0.001
Neutrophil, Mean ± SD	79.58 ± 14.25	79.19 ± 13.89	80.70 ± 15.19	*t* = −1.840	0.066
Lymphocytes, *M* (*Q*_1_, *Q*_3_)	9.00 (5.00,15.40)	10.00 (5.70,16.50)	6.80 (3.90,11.60)	*Z* = −8.188	<0.001
TBIL, *M* (*Q*_1_, *Q*_3_)	0.60 (0.40,1.10)	0.60 (0.40,1.00)	0.61 (0.40,1.40)	*Z* = 2.843	0.004
Hematocrit, Mean ± SD	33.29 ± 6.16	33.36 ± 6.08	33.09 ± 6.39	*t* = 0.780	0.438
RDW, Mean ± SD	15.95 ± 2.54	15.72 ± 2.46	16.60 ± 2.68	*t* = −6.090	<0.001

CKD, chronic kidney disease; PaO_2_/FiO_2_, partial arterial oxygen pressure/the fraction of inspired oxygen; SAPS II, the Simplified Acute Physiology Score II; GCS, Glasgow Coma Scale; SOFA, sequential organ failure assessment; SBP, systolic blood pressure; MAP, mean arterial pressure; RBC, red blood count; PLT: platelets; INR, international normalized ratio; WBC, white blood cell count; BUN, blood urea nitrogen; TBIL, total bilirubin; RDW, red cell distribution width.

### Predictors influencing the mortality in ICU patients with metastatic cancer

**Figure 2:**
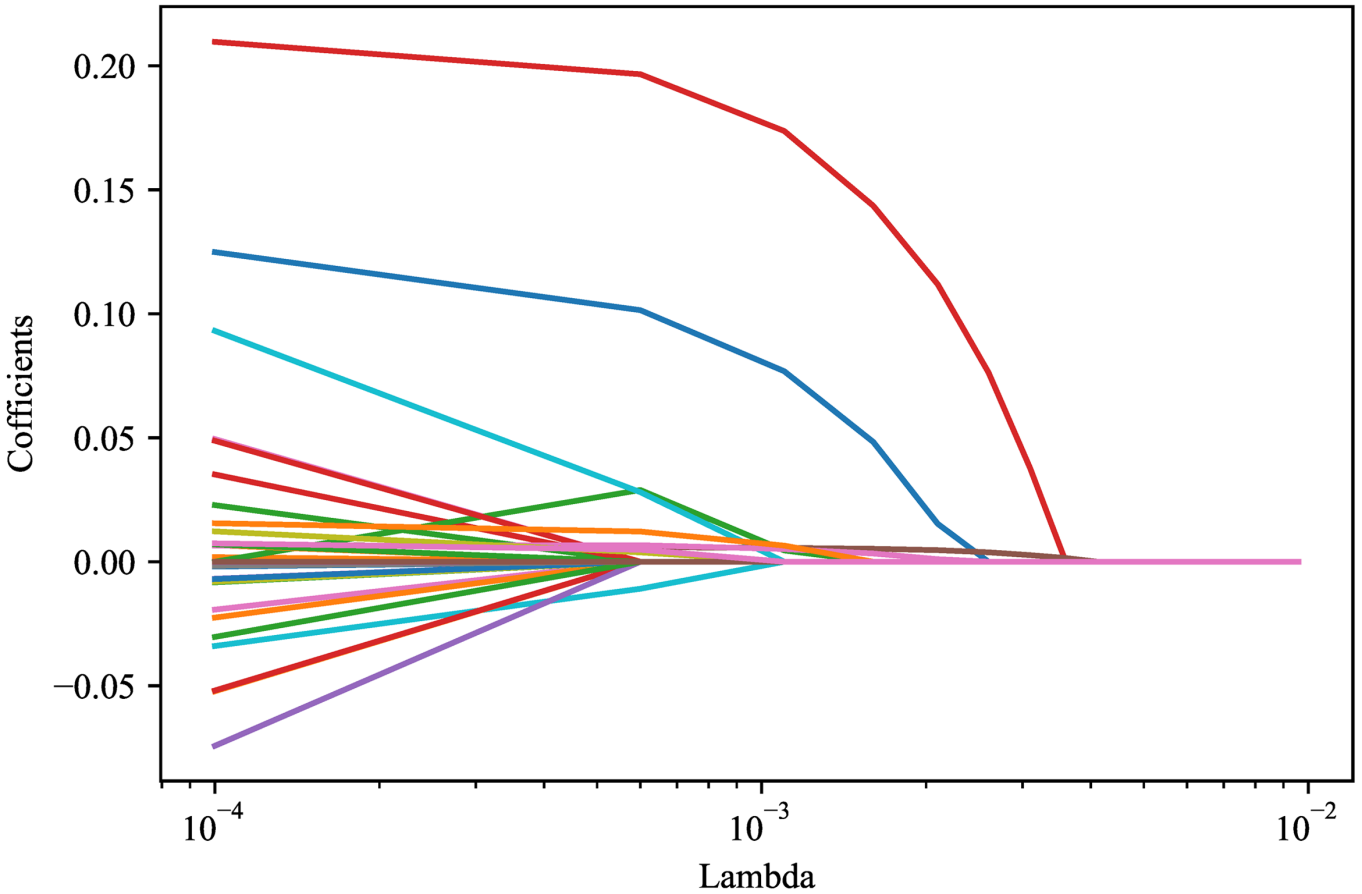
LASSO regression analysis screening the predictors for in-hospital mortality of metastasitc cancer patients.

LASSO regression analysis was applied to screen the predictors for the mortality in ICU patients with metastatic cancer. As variables including ventilation, PaO_2_/FiO_2_, PLT, TBIL, and creatinine were included in SOFA score, and variables including heart rate, SBP, temperature, ventilation, PaO_2_/FiO_2_, BUN, WBC, potassium, sodium, bicarbonate, and TBIL were involved in SAPS II, these variables were not included in LASSO regression to avoid collinearity. The results depicted that age, respiratory failure, SOFA score, SAPS II, glucose, RDW and lactate were predictors of in-hospital death in patients with metastatic cancer in ICUs (Figure 2). Multicollinearity analysis reported that the variance inflation factor (VIF) of these predictors were <5 (Supplementary Table S3), indicating low collinearity in these variables. The final equation for the prediction model was ln(*P*/(1 + *P*)) = −5.9830 + 0.0174 × age + 1.3686 × respiratory failure + 0.0537 × SAPS II + 0.0312 × SOFA + 0.1278 × lactate − 0.0026 × glucose + 0.0772 × RDW.

### Construction and validation of the prediction model

**Figure 3:**
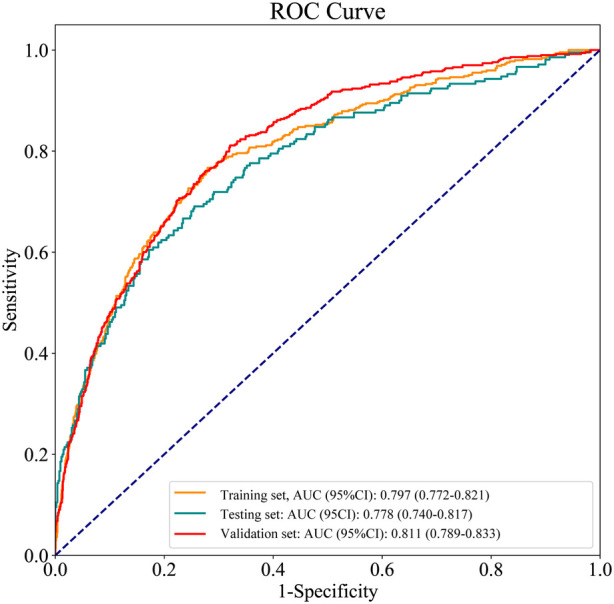
ROC curves for the prediction model in the training set, testing set and validation set.

**Figure 4:**
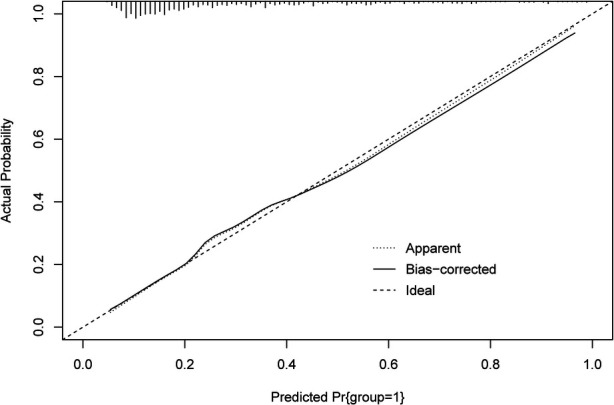
The calibration curves of model in the testing set.

**Figure 5:**
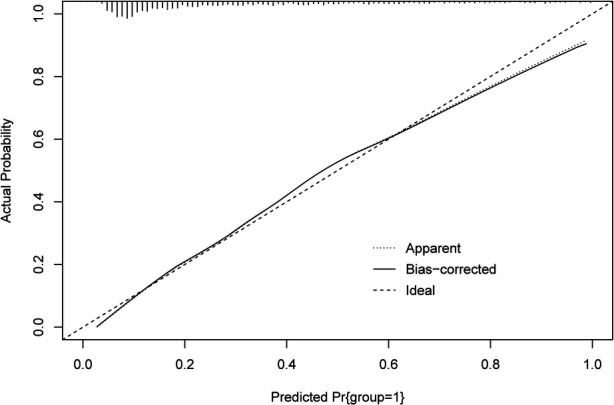
The calibration curves of the model in the validation set.

**Figure 6:**
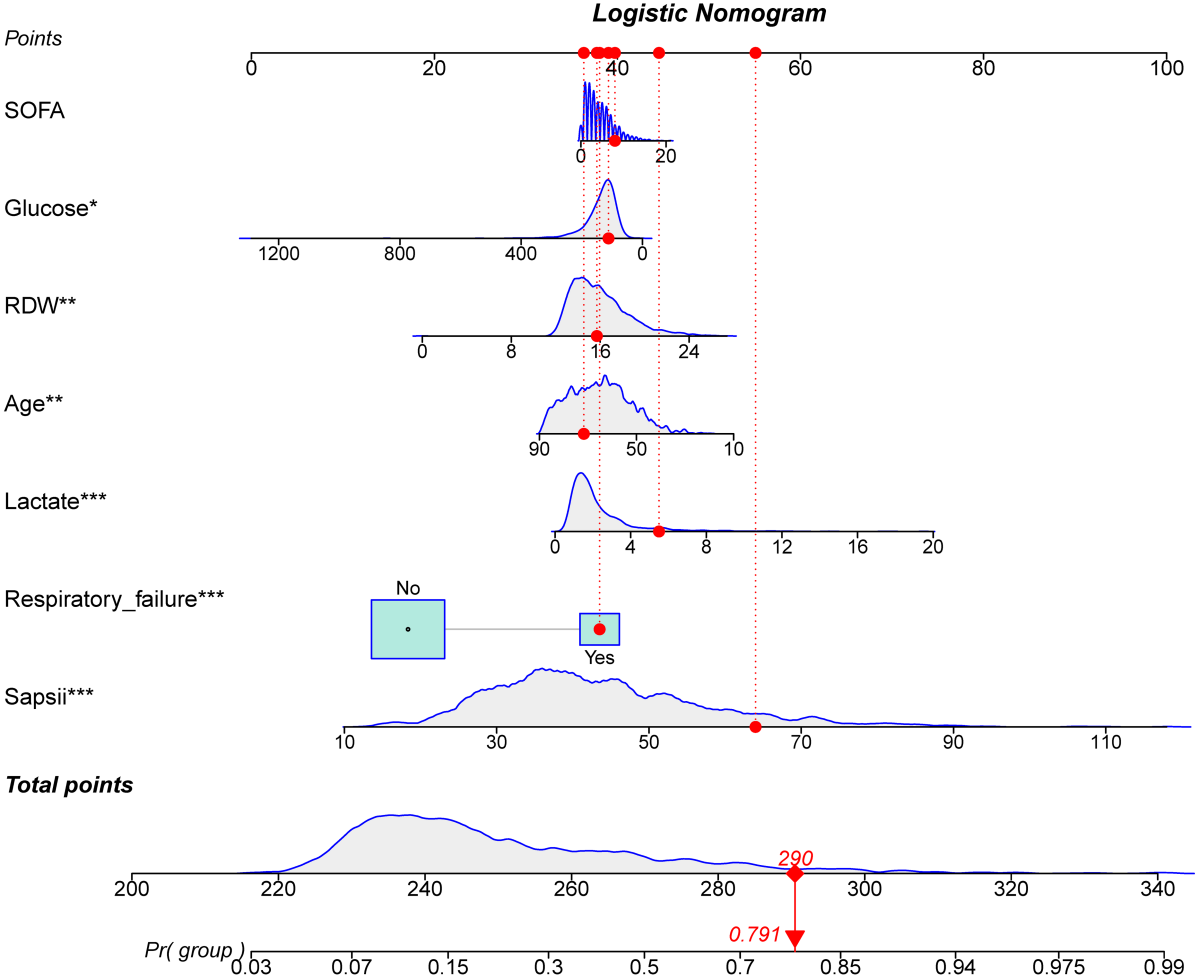
The nomogram for predicting the probability of death in patients with metastatic cancer.

The ROC and calibration curves were applied for evaluating the predictive value of the prediction model. The data delineated that in the training set, the AUC was 0.797 (95% CI, 0.776–0.825), the sensitivity was 0.767 (95% CI, 0.728–0.806), the specificity was 0.720 (95% CI, 0.696–0.745), and the NPV was 0.898 (95% CI, 0.880–0.917). In the testing set, the AUC was 0.778 (95% CI, 0.740–0.817), the sensitivity was 0.690 (95% CI, 0.628–0.753), the specificity was 0.745 (95% CI, 0.708–0.782), and the NPV was 0.858 (95% CI, 0.826–0.890) (Table 3). The data from MIMIC-IV were applied as a validation set, and the detailed characteristics of participants were shown in Supplementary Table S4. The AUC in the validation set was 0.811 (95% CI, 0.789–0.833), the sensitivity was 0.811 (95 %CI, 0.777–0.846), the specificity was 0.679 (95% CI, 0.653–0.705) and the NPV was 0.899 (95% CI, 0.879–0.918) (Table 3) (Figure 3). The calibration curves of the testing set (Figure 4) and the validation set (Figure 5) were plotted, which depicted that the prediction values of the model deviated slightly from the ideal model, but was close to matching, indicating the prediction model had good agreement between the predictive probability and the actual probability. A nomogram was plotted and a sample was randomly selected from the participants to validate the prediction ability of our model. The values of each predictor were represented by the red dot in the Figure 6. Each value corresponded to the score in the first row, and then the total score of the predictors in the sample was 290 in the penultimate row, and the probability of death in the sample was 0.791, which was the same with the actual outcome.

**Table 3:** Construction and validation of the prediction model.

Parameter	Database		
	Training set (95% CI)	Testing set (95% CI)	Validation set (95% CI)
AUC (95% CI)	0.797 (0.772–0.821)	0.778 (0.740–0.817)	0.811 (0.789–0.833)
Sensitivity (95% CI)	0.767 (0.728–0.806)	0.690 (0.628–0.753)	0.811 (0.777–0.846)
Specificity (95% CI)	0.720 (0.696–0.745)	0.745 (0.708–0.782)	0.679 (0.653–0.705)
PPV (95% CI)	0.489 (0.452–0.526)	0.518 (0.459–0.576)	0.506 (0.472–0.541)
NPV (95% CI)	0.898 (0.880–0.917)	0.858 (0.826–0.890)	0.899 (0.879–0.918)

AUC, area under the curve; OR, odds ratio; CI, confidence interval.

### Evaluation of the prediction value of the model in different cancers

**Figure 7:**
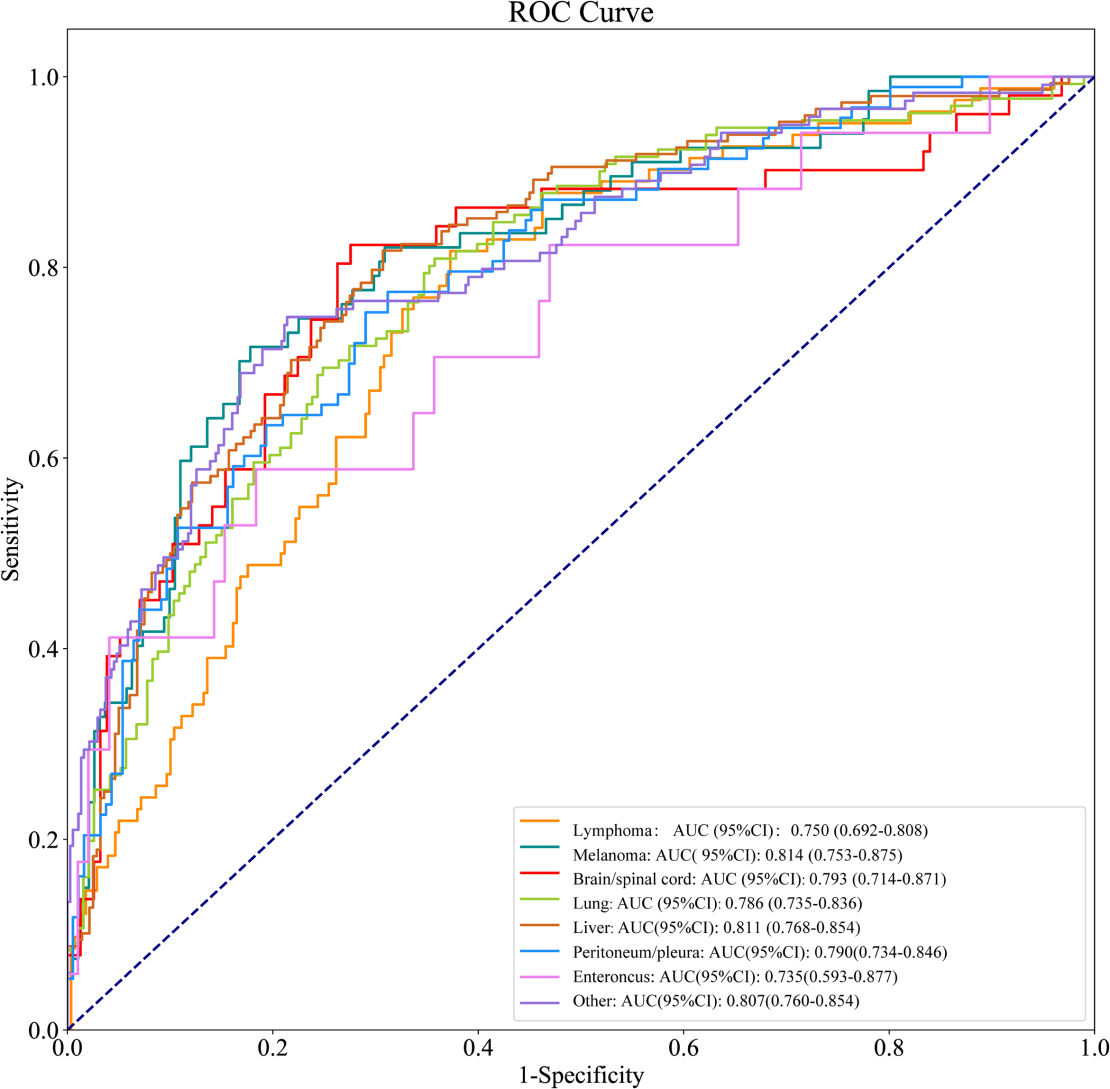
The nomogram for predicting the probabilityof death in patients with metastatic cancer.

As shown in Table 4, the AUCs of the prediction model for lymphoma, myeloma, brain/spinal cord, lung, liver, peritoneum/pleura, enteroncus and other cancer populations were 0.750 (95% CI, 0.692–0.808), 0.814 (95% CI, 0.753–0.875), 0.793 (95% CI, 0.714–0.871), 0.786 (95% CI, 0.735–0.836), 0.811 (95% CI, 0.768–0.854), 0.790 (95% CI, 0.734–0.846), 0.735 (95% CI, 0.593–0.877) and 0.807 (95% CI, 0.760–0.854), respectively (Figure 7). The sensitivity and specificity of the prediction models for different cancer types were exhibited in Table 4.

**Table 4:** Evaluation of the prediction value of the model in different cancers.

Cancer	Samples in MIMIC-III and MIMIC-IV	AUC (95% CI)	Sensitivity (95% CI)	Specificity (95% CI)
Lymphoa	361	0.750 (0.692–0.808)	0.817 (0.733–0.901)	0.627 (0.571–0.684)
Myeloma	258	0.814 (0.753–0.875)	0.716 (0.608–0.824)	0.822 (0.768–0.876)
Brain/spinal cord	207	0.793 (0.714–0.871)	0.824 (0.719–0.928)	0.724 (0.654–0.794)
Lung	324	0.786 (0.735–0.836)	0.809 (0.742–0.876)	0.642 (0.575–0.710)
Liver	428	0.811 (0.768–0.854)	0.818 (0.755–0.880)	0.693 (0.639–0.747)
Peritoneum/pleura	279	0.790 (0.734–0.846)	0.753 (0.665–0.840)	0.710 (0.644–0.775)
Enteroncus	115	0.735 (0.593–0.877)	0.588 (0.354–0.822)	0.816 (0.740–0.893)
Other	493	0.807 (0.760–0.854)	0.748 (0.670–0.826)	0.786 (0.745–0.828)

AUC, area under the curve; CI, confidence interval.

## Discussion

The presented study established a prediction model for in-hospital mortality in metastasis patients using the data of 2,462 patients from the MIMIC-III and validated the performance of the prediction model through the data of 1,726 patients from MIMIC-IV. The results showed that the prediction model constructed in our study presented good predictive ability for the in-hospital mortality in patients with metastatic cancer in ICUs based on the predictors including age, respiratory failure, SOFA score, SAPS II, glucose, RDW and lactate. The model also depicted good predictive performance in different cancer types. The findings of this study might help identify patients with metastatic cancer who were at high risk of death in hospital, and reminded the clinicians to make proper interventions on these patients and improve their outcomes.

The prediction model for the mortality in metastatic cancer patients in ICUs was established. Compared with the previous prediction model from Lee Cheng et al. (11), our model was only included patients with metastatic cancer, and patients with advanced cancer but without metastasis were not included in this study, this could decrease the inclusion bias in patients. As the characteristics of patients with metastatic or advanced cancers were not the same (12). Some patients were at advanced stage but without metastasis (16). The model was more targeted for predicting the mortality in patients with metastatic cancer in ICUs. In addition, the predictive value of our model was higher than the previous model (AUC: 0.797 vs. 0.74). The calibration curve revealed that the prediction model was well calibrated. The sensitivity, specificity, PPV, NPV were also assessed to more comprehensively estimate the discriminatory ability of the model (17). The sensitivity, specificity, PPV, NPV of our model were high, which indicated the good predictive value of the model. The predictive value of the model was also validated in the testing set, and it also showed good discriminatory ability. External validation of the predictive performance was performed using the data from MIMIC-IV, and the results indicated good predictive value of the model. We also verified the prediction values of the model in different types of cancers, the predictive performances were all very good in different cancers. This suggested that our model was suitable for predicting the mortality in patients with different metastatic cancers. A nomogram was plotted, which can easily obtain the probability of death in patients with metastatic cancer in ICUs. In addition, an online tool was established based on our model to help the clinicians to quickly identify patients with high risk of mortality (https://wumeizhen.shinyapps.io/DynNomapp/). The findings of our model can help clinicians quickly identify metastatic cancer patients in ICUs with high risk of in-hospital death. Previous studies demonstrated that cancer patients in ICUs may derive benefits from different intensive care support (18), and if patients were predicted at high risk of mortality, clinicians should make timely interventions on their treatments and ICU management to improve their outcomes.

Acute respiratory failure is the major cause for ICU admission and death in patients with cancer (19, 20). Several studies have indicated that the in-hospital mortality of patients receiving open repair of descending thoracic aortic aneurysm with respiratory failure was higher than patients without respiratory failure (21). Another study also demonstrated that respiratory failure increased the hospital morality of lung cancer patients in ICUs by 3.00 times, which was an independent risk factor for hospital morality of lung cancer patients in ICUs (22). These findings were allied with the results in our study, which revealed that respiratory failure was associated with the risk of mortality in patients with metastatic cancer. For participants with respiratory failure, special care and early treatments should be provided. The SOFA score assesses the organ dysfunction over a period of time and evaluates the morbidity in ICU patients based on a scale of 0–4 for each organ system (23). The SOFA score was reported to be a sensitive and accurate tool for predicting the mortality in cancer patients admitted to ICUs with suspected infection (24). In our study, SOFA score was correlated with the risk of death in patients with metastatic cancer.

In critically ill patients with cancer, high levels of lactate may represent the cancer disease itself and the metabolism of the cancer cells, that is the Warburg effect (25). High levels of lactate was identified in some cancers, such as the aerobic glycolysis, the tumor cells consume glucose in the presence of oxygen, and product high levels of lactate, resulting in lactic acidosis (26). Additionally, increased levels of lactate were reported to be an essential predictor for the mortality in patients with cancer (26, 27). These findings gave support to the data in this study, delineating that the levels of lactate were associated with the risk of death in metastatic cancer patients in ICUs. For those with high levels of lactate, interventions such as sodium bicarbonate should be applied if necessary. Previous studies indicated that age was an important prognostic index of metastatic cancer patients, and patients with old ages might benefit less from the treatment and was associated with a higher risk of mortality (28, 29). Herein, age was also included as a predictor for the mortality in metastatic cancer patients in ICUs. RDW is reported to be of great value as a marker of poor prognosis for different cancers (30–33). The increased value of RDW might reflect the imbalance of RBC homeostasis, which may be due to the impaired RBC formation ability and abnormal RBC survival caused by the body's abnormal metabolism (34). In our study, RDW was identified to be a predictor for the risk of mortality in metastatic cancer patients. Special treatments might be required to patients with abnormal RDW.

Several limitations existed in the current study. Firstly, the data of included patients were extracted from the MIMIC-III database, and some important information such as the primary site of the tumors and the detailed treatment before admitted to or during ICUs. Secondly, the external validation of our model was not performed. Thirdly, the patients from MIMIC-III were mainly White people, whether the prediction model was suitable for patients from other countries still needs more verification. In the future, more well-designed prospective studies were required to validate the results in the present study.

## Conclusions

In this study, the data of 2,462 patients were collected from the MIMIC-III to construct the prediction model for the in-hospital mortality of ICU patients with metastatic cancer and the data of 1,726 patients from MIMIC-IV were applied to validate the performance of the model. The model exhibited good predictive ability for the in-hospital mortality in patients with metastatic cancer in ICUs and also showed good predictive value for patients with different cancers. An online tool was made to help the clinicians to quickly use the model. The findings of the current study might help identify patients with metastatic cancer who were at high risk of death in hospital, and the clinicians should make timely interventions on these patients and improve their outcomes.

## Data Availability

Publicly available datasets were analyzed in this study. This data can be found here: MIMIC-III database, https://mimic.physionet.org/iii/.
