# Supplementary material for: A prediction model for in-hospital mortality in intensive care unit patients with metastatic cancer
Source: Front Surg. 2023 Jan 30;10:992936. doi: 10.3389/fsurg.2023.992936 (PMC9922743; doi:10.3389/fsurg.2023.992936)
Supplement: Supplementary file 1 [file Datasheet1.docx]

Supplementary Table 1 The ICD-9 code of different cancers

| ICD9 code | Sample | Long title |
| --- | --- | --- |
| 1960 | 53 | Secondary and unspecified malignant neoplasm of lymph nodes of head,face,and neck |
| 1961 | 112 | Secondary and unspecified malignant neoplasm of intrathoracic lymph nodes |
| 1962 | 121 | Secondary and unspecified malignant neoplasm of intra-abdominal lymph nodes |
| 1963 | 11 | Secondary and unspecified malignant neoplasm of lymph nodes of axilla and upper limb |
| 1965 | 6 | Secondary and unspecified malignant neoplasm of lymph nodes of inguinal region and lower limb |
| 1966 | 8 | Secondary and unspecified malignant neoplasm of intrapelvic lymph nodes |
| 1968 | 9 | Secondary and unspecified malignant neoplasm of lymph nodes of multiple sites |
| 1969 | 3 | Secondary and unspecified malignant neoplasm of lymph nodes,site unspecified |
| 1970 | 273 | Secondary malignant neoplasm oflung |
| 1971 | 20 | Secondary malignant neoplasm of mediastinum |
| 1972 | 104 | Secondary malignant neoplasm of pleura |
| 1973 | 15 | Secondary malignant neoplasm of other respiratory organs |
| 1974 | 43 | Secondary malignant neoplasm of small intestine including duodenum |
| 1975 | 16 | Secondary malignant neoplasm of large intestine and rectum |
| 1976 | 121 | Secondary malignant neoplasm of retroperitoneum and peritoneum |
| 1977 | 328 | Malignant neoplasm of liver,secondary |
| 1978 | 44 | Secondary malignant neoplasm of other digestive organs and spleen |
| 1980 | 4 | Secondary malignant neoplasm of kidney |
| 1981 | 7 | Secondary malignant neoplasm of other urinary organs Secondary malignant neoplasm of skin |
| 1982 | 9 | Secondary malignant neoplasm of skin |
| 1983 | 376 | Secondary malignant neoplasm of brain and spinal cord |
| 1984 | 26 | Secondary malignant neoplasm of other parts of nervous system |
| 1985 | 325 | Secondary malignant neoplasm of bone and bone marrow |
| 1986 | 7 | Secondary malignant neoplasm of ovary |
| 1987 | 22 | Secondary malignant neoplasm of adrenal gland |
| 19882 | 42 | Secondary malignant neoplasm of genital organs |
| 19889 | 13 | Secondary malignant neoplasm of other specified sites |
| 1991 | 141 | Other malignant neoplasm without specification of site |

ICD-9: the International Classification of Disease 9th Edition

Supplementary Table 2 The missing values and sensitivity analysis

between the data before and after manipulation

| Variable | Missing (%) | Before manipulation | After manipulation | Statistical magnitude | *P* |
| --- | --- | --- | --- | --- | --- |
| Gender | 0.0 |  |  |  |  |
| Age | 0.0 |  |  |  |  |
| Insurance | 0.0 |  |  |  |  |
| Congestive heart failure | 0.0 |  |  |  |  |
| Cardiac dysrhythmia | 0.0 |  |  |  |  |
| Respiratory failure | 0.0 |  |  |  |  |
| Atrial fibrillation | 0.0 |  |  |  |  |
| CKD | 0.0 |  |  |  |  |
| Myocardial infarction | 0.0 |  |  |  |  |
| Diabetes mellitus | 0.0 |  |  |  |  |
| Hypertension | 0.0 |  |  |  |  |
| Hyperlipidemia | 0.0 |  |  |  |  |
| First care unit | 0.0 |  |  |  |  |
| Septicemia | 0.0 |  |  |  |  |
| Tumor type | 0.0 |  |  |  |  |
| Surgery | 0.0 |  |  |  |  |
| Chemotherapy | 0.0 |  |  |  |  |
| Radiotherapy | 0.0 |  |  |  |  |
| Length of hospital | 0.0 |  |  |  |  |
| Length of ICU | 0.0 |  |  |  |  |
| PaO_2_/FiO_2_ | 0.21 | 333.33 (234.00,422.50) | 324.64 (217.75,422.25) | Z=-1.495 | 0.135 |
| Albumin | 0.19 | 3.05±0.67 | 3.00±0.67 | t=0.260 | 0.722 |
| TBIL | 0.18 | 0.60 (0.40,1.10) | 0.60 (0.40,1.20) | Z=0.445 | 0.432 |
| Lactate | 0.13 | 1.70 (1.30,2.60) | 1.80 (1.30,2.60) | Z=0.844 | 0.399 |
| pH | 0.16 | 7.39±0.09 | 7.38±0.10 | t=1.200 | 0.145 |
| Neutrophil | 0.18 | 79.39±14.63 | 79.12±15.07 | t=0.610 | 0.540 |
| Lymphocytes | 0.18 | 9.00 (5.00,15.60) | 9.00 (5.00,15.50) | Z=-0.780 | 0.435 |
| SBP | 0.08 | 125.16±22.34 | 126.32±21.92 | t=-1.800 | 0.071 |
| Race | 0.06 |  |  | χ^2^=0.031 | 1.000 |
| White |  | 1996 (81.07) | 1873 (81.19) |  |  |
| Black |  | 201 (8.16) | 188 (8.15) |  |  |
| Asian |  | 114 (4.63) | 107 (4.64) |  |  |
| Hispanic |  | 58 (2.36) | 54 (2.34) |  |  |
| Others |  | 93 (3.78) | 85 (3.68) |  |  |
| Heart Rate | 0.04 | 96.13±20.32 | 96.41±20.35 | t=-0.470 | 0.637 |
| INR | 0.04 | 1.20 (1.10,1.40) | 1.20 (1.10,1.40) | Z=-0.131 | 0.896 |
| Temperature | 0.05 | 36.73±1.90 | 36.74±1.93 | t=-0.160 | 0.872 |
| DBP | 0.05 | 66.03±14.90 | 66.71±14.64 | t=-1.590 | 0.111 |
| Marital | 0.03 |  |  | χ^2^=0.031 | 0.999 |
| Married |  | 1504 (61.09) | 1462 (61.15) |  |  |
| Single |  | 500 (20.31) | 488 (20.41) |  |  |
| Widowed |  | 290 (11.78) | 278 (11.63) |  |  |
| Divorced/separated |  | 168 (6.82) | 163 (6.82) |  |  |
| GCS score | 0.03 | 12.15±3.83 | 12.12±3.84 | t=0.360 | 0.721 |
| Phosphate | 0.02 | 3.50 (2.90,4.20) | 3.50 (2.90,4.20) | Z=-0.110 | 0.913 |
| Calcium | 0.02 | 8.61±1.03 | 8.62±1.03 | t=-0.080 | 0.940 |
| Ventilation | 0.02 | 642 (26.08) | 639 (26.46) | χ^2^=0.092 | 0.761 |
| MAP | 0.01 | 82.53±17.86 | 82.39±17.49 | t=0.270 | 0.788 |
| WBC | 0.01 | 10.20 (7.10,14.70) | 10.20 (7.10,14.70) | Z=0.022 | 0.982 |
| Glucose | 0.01 | 126.00 (104.00,157.00) | 126.00 (104.00,157.00) | Z=-0.018 | 0.986 |
| Sodium | 0.01 | 136.70±5.25 | 136.70±5.24 | t=-0.000 | 0.999 |
| Bicarbonate | 0.01 | 24.18±4.70 | 24.19±4.69 | t=-0.060 | 0.955 |
| RBC | 0.01 | 3.74±0.75 | 3.74±0.75 | t=0.050 | 0.961 |
| Hemoglobin | 0.01 | 11.05±2.20 | 11.05±2.20 | t=0.050 | 0.959 |
| RDW | 0.01 | 15.96±2.55 | 15.97±2.55 | t=-0.030 | 0.977 |
| Potassium | 0.01 | 4.35±0.85 | 4.35±0.85 | t=0.110 | 0.909 |
| PLT | 0.01 | 259.00 (179.00,363.00) | 260.00 (180.00,363.00) | Z=0.074 | 0.941 |
| Creatinine | 0.01 | 0.90 (0.70,1.30) | 0.90 (0.70,1.30) | Z=-0.002 | 0.998 |
| BUN | 0.01 | 20.00 (14.00,30.00) | 20.00 (14.00,30.00) | Z=0.087 | 0.931 |
| Hematocrit | 0.01 | 33.15±6.22 | 33.15±6.23 | t=0.040 | 0.967 |
| SOFA score | 0.01 | 4.00 (2.00,6.00) | 4.00 (2.00,6.00) | Z=0.079 | 0.937 |

CKD: chronic kidney disease, ICU: intensive care unit, PaO_2_/FiO_2_: partial arterial oxygen pressure/the fraction of inspired oxygen, GCS: Glasgow Coma Scale, SOFA: sequential organ failure assessment, SBP: systolic blood pressure, MAP: mean arterial pressure, RBC: red blood count, PLT: platelets, pH: pondus hydrogenii, WBC: white blood cell count, BUN: blood urea nitrogen, TBIL: total bilirubin

Supplementary Table 3 Multicollinearity analysis of the VIF of predictors

| Variables | VIF |
| --- | --- |
| Age | 1.326839 |
| Respiratory failure | 1.117709 |
| SAPSII score | 2.611576 |
| SOFA score | 2.183699 |
| Lactate | 1.180611 |
| Glucose | 1.042979 |
| RDW | 1.049977 |

VIF: variance inflation factor, SAPS II: the Simplified Acute Physiology Score II, SOFA: the sequential organ failure assessment

Supplementary Table 4 The baseline characteristics of participants from MIMIC-IV

|  | | Group | |  | |
| --- | --- | --- | --- | --- | --- |
| Variables | Total (n=1726) | Survival group (n=1228) | Death group (n=498) | Statistics | *P* |
| Age, Mean±SD | 65.68 ± 12.31 | 65.59 ± 12.17 | 65.91 ± 12.63 | t=-0.48 | 0.632 |
| Gender, n (%) |  |  |  | χ^2^=0.014 | 0.906 |
| Female | 776 (44.96) | 551 (44.87) | 225 (45.18) |  |  |
| Male | 950 (55.04) | 677 (55.13) | 273 (54.82) |  |  |
| Race, n (%) |  |  |  | χ^2^=12.194 | 0.016 |
| White | 1209 (70.05) | 882 (71.82) | 327 (65.66) |  |  |
| Black | 185 (10.72) | 115 (9.36) | 70 (14.06) |  |  |
| Asian | 101 (5.85) | 73 (5.94) | 28 (5.62) |  |  |
| Hispanic | 44 (2.55) | 34 (2.77) | 10 (2.01) |  |  |
| Others | 187 (10.83) | 124 (10.10) | 63 (12.65) |  |  |
| Marital, n (%) |  |  |  | χ^2^=0.637 | 0.888 |
| Married | 946 (54.81) | 666 (54.23) | 280 (56.22) |  |  |
| Single | 421 (24.39) | 305 (24.84) | 116 (23.29) |  |  |
| Widowed | 194 (11.24) | 139 (11.32) | 55 (11.04) |  |  |
| Divorced/separated | 165 (9.56) | 118 (9.61) | 47 (9.44) |  |  |
| Congestive heart failure, n (%) |  |  |  | χ^2^=0.003 | 0.956 |
| No | 1437 (83.26) | 1022 (83.22) | 415 (83.33) |  |  |
| Yes | 289 (16.74) | 206 (16.78) | 83 (16.67) |  |  |
| Cardiac dysrhythmia, n (%) |  |  |  | χ^2^=4.035 | 0.045 |
| No | 1393 (80.71) | 1006 (81.92) | 387 (77.71) |  |  |
| Yes | 333 (19.29) | 222 (18.08) | 111 (22.29) |  |  |
| Respiratory failure, n (%) |  |  |  | χ^2^=201.290 | <.001 |
| No | 1091 (63.21) | 905 (73.70) | 186 (37.35) |  |  |
| Yes | 635 (36.79) | 323 (26.30) | 312 (62.65) |  |  |
| Atrial fibrillation, n (%) |  |  |  | χ^2^=3.000 | 0.083 |
| No | 1406 (81.46) | 1013 (82.49) | 393 (78.92) |  |  |
| Yes | 320 (18.54) | 215 (17.51) | 105 (21.08) |  |  |
| CKD, n (%) |  |  |  | χ^2^=2.731 | 0.098 |
| No | 1473 (85.34) | 1059 (86.24) | 414 (83.13) |  |  |
| Yes | 253 (14.66) | 169 (13.76) | 84 (16.87) |  |  |
| Myocardial infarction, n (%) |  |  |  | χ^2^=2.806 | 0.094 |
| No | 1535 (88.93) | 1102 (89.74) | 433 (86.95) |  |  |
| Yes | 191 (11.07) | 126 (10.26) | 65 (13.05) |  |  |
| Diabetes mellitus, n (%) |  |  |  | χ^2^=0.196 | 0.658 |
| No | 1288 (74.62) | 920 (74.92) | 368 (73.90) |  |  |
| Yes | 438 (25.38) | 308 (25.08) | 130 (26.10) |  |  |
| Hypertension, n (%) |  |  |  | χ^2^=2.053 | 0.152 |
| No | 844 (48.90) | 587 (47.80) | 257 (51.61) |  |  |
| Yes | 882 (51.10) | 641 (52.20) | 241 (48.39) |  |  |
| Hyperlipidemia, n (%) |  |  |  | χ^2^=2.148 | 0.143 |
| No | 1218 (70.57) | 854 (69.54) | 364 (73.09) |  |  |
| Yes | 508 (29.43) | 374 (30.46) | 134 (26.91) |  |  |
| Sepsis, n (%) |  |  |  | χ^2^=36.243 | <.001 |
| No | 1515 (87.78) | 1115 (90.80) | 400 (80.32) |  |  |
| Yes | 211 (12.22) | 113 (9.20) | 98 (19.68) |  |  |
| Tumor type, n (%) |  |  |  | χ^2^=46.275 | <.001 |
| Lymphoma | 264 (15.30) | 200 (16.29) | 64 (12.85) |  |  |
| Myeloma | 165 (9.56) | 123 (10.02) | 42 (8.43) |  |  |
| Brain/spinal cord | 102 (5.91) | 76 (6.19) | 26 (5.22) |  |  |
| Lung | 242 (14.02) | 142 (11.56) | 100 (20.08) |  |  |
| Liver | 326 (18.89) | 216 (17.59) | 110 (22.09) |  |  |
| Peritoneum/pleura | 198 (11.47) | 131 (10.67) | 67 (13.45) |  |  |
| Enteroncus | 83 (4.81) | 73 (5.94) | 10 (2.01) |  |  |
| Other | 346 (20.05) | 267 (21.74) | 79 (15.86) |  |  |
| Surgery, n (%) |  |  |  | χ^2^=0.611 | 0.435 |
| No | 666 (38.59) | 481 (39.17) | 185 (37.15) |  |  |
| Yes | 1060 (61.41) | 747 (60.83) | 313 (62.85) |  |  |
| Chemotherapy, n (%) |  |  |  | χ^2^=0.673 | 0.412 |
| No | 1683 (97.51) | 1195 (97.31) | 488 (97.99) |  |  |
| Yes | 43 (2.49) | 33 (2.69) | 10 (2.01) |  |  |
| Radiotherapy, n (%) |  |  |  | - | 0.109 |
| No | 1709 (99.02) | 1219 (99.27) | 490 (98.39) |  |  |
| Yes | 17 (0.98) | 9 (0.73) | 8 (1.61) |  |  |
| Length of hospital, M (Q_1_,Q_3_) | 8.00 (5.00,14.00) | 9.00 (5.50,15.00) | 6.00 (2.00,12.00) | Z=-8.954 | <.001 |
| Length of ICU, M (Q_1_,Q_3_) | 2.24 (1.39,4.19) | 2.11 (1.31,3.86) | 2.84 (1.44,5.21) | Z=4.136 | <.001 |
| Ventilation, n (%) |  |  |  | χ^2^=22.379 | <.001 |
| No | 313 (18.13) | 257 (20.93) | 56 (11.24) |  |  |
| Yes | 1413 (81.87) | 971 (79.07) | 442 (88.76) |  |  |
| PaO_2_/FiO_2_, M (Q_1_,Q_3_) | 2.40 (1.46,3.70) | 2.78 (1.74,3.98) | 1.74 (1.03,2.80) | Z=-7.241 | <.001 |
| SAPS II score, Mean±SD | 48.07 ± 14.10 | 44.51 ± 11.38 | 56.85 ± 16.18 | t=-15.54 | <.001 |
| GCS score, M (Q_1_,Q_3_) | 14.00 (11.00,15.00) | 14.00 (13.00,15.00) | 13.00 (7.00,15.00) | Z=-10.618 | <.001 |
| SOFA score, M (Q_1_,Q_3_) | 5.00 (3.00,8.00) | 4.00 (3.00,7.00) | 8.00 (6.00,11.00) | Z=17.065 | <.001 |
| Temperature, Mean±SD | 36.73 ± 0.78 | 36.75 ± 0.76 | 36.67 ± 0.81 | t=1.89 | 0.059 |
| Heart Rate, Mean±SD | 97.30 ± 21.50 | 94.86 ± 21.01 | 103.33 ± 21.54 | t=-7.53 | <.001 |
| SBP, Mean±SD | 119.26 ± 24.89 | 120.10 ± 25.07 | 117.17 ± 24.33 | t=2.22 | 0.027 |
| DBP, Mean±SD | 67.12 ± 17.36 | 66.79 ± 16.62 | 67.94 ± 19.05 | t=-1.18 | 0.240 |
| MAP, Mean±SD | 81.02 ± 19.79 | 81.47 ± 19.68 | 79.91 ± 20.03 | t=1.48 | 0.138 |
| RBC, Mean±SD | 3.39 ± 0.73 | 3.42 ± 0.73 | 3.32 ± 0.73 | t=2.58 | 0.010 |
| Sodium, Mean±SD | 136.62 ± 5.31 | 136.66 ± 5.06 | 136.51 ± 5.90 | t=0.52 | 0.606 |
| Potassium, Mean±SD | 4.26 ± 0.80 | 4.21 ± 0.77 | 4.39 ± 0.85 | t=-4.07 | <.001 |
| Phosphate, M (Q_1_,Q_3_) | 3.70 (2.90,4.60) | 3.60 (2.80,4.40) | 4.00 (3.10,5.00) | Z=5.508 | <.001 |
| Calcium, Mean±SD | 7.90 (7.20,8.60) | 8.00 (7.30,8.60) | 7.90 (7.20,8.60) | Z=-0.353 | 0.724 |
| PLT, M (Q_1_,Q_3_) | 209.00 (137.00,297.00) | 212.00 (144.00,294.00) | 200.50 (112.00,312.00) | Z=-2.005 | 0.045 |
| pH, Mean±SD | 7.35 ± 0.10 | 7.37 ± 0.09 | 7.32 ± 0.13 | t=8.58 | <.001 |
| Lactate, M (Q_1_,Q_3_) | 1.80 (1.30,2.90) | 1.70 (1.20,2.60) | 2.20 (1.50,3.90) | Z=8.733 | <.001 |
| INR, M (Q_1_,Q_3_) | 1.30 (1.20,1.60) | 1.30 (1.20,1.50) | 1.40 (1.30,1.90) | Z=8.137 | <.001 |
| Albumin, Mean±SD | 2.73 ± 0.61 | 2.81 ± 0.61 | 2.59 ± 0.58 | t=4.45 | <.001 |
| Hemoglobin, Mean±SD | 9.90 ± 2.09 | 9.98 ± 2.08 | 9.71 ± 2.10 | t=2.42 | 0.016 |
| WBC, M (Q_1_,Q_3_) | 11.40 (7.20,16.70) | 10.85 (7.10,15.70) | 13.20 (7.90,18.90) | Z=4.081 | <.001 |
| Glucose, M (Q_1_,Q_3_) | 126.50 (102.00,165.00) | 127.00 (105.00,165.00) | 124.00 (97.00,163.00) | Z=-2.506 | 0.012 |
| Creatinine, M (Q_1_,Q_3_) | 1.00 (0.70,1.50) | 0.90 (0.70,1.40) | 1.20 (0.70,1.90) | Z=6.171 | <.001 |
| BUN, M (Q_1_,Q_3_) | 21.00 (14.00,35.00) | 19.00 (13.00,30.00) | 28.00 (18.00,47.00) | Z=9.533 | <.001 |
| Bicarbonate, Mean±SD | 21.54 ± 5.08 | 21.98 ± 4.69 | 20.47 ± 5.81 | t=5.15 | <.001 |
| Neutrophil, Mean±SD | 79.10 ± 16.84 | 79.25 ± 16.73 | 78.82 ± 17.07 | t=0.33 | 0.743 |
| Lymphocytes, M (Q_1_,Q_3_) | 5.40 (2.00,10.60) | 5.60 (2.00,11.00) | 5.00 (2.00,9.20) | Z=-0.816 | 0.414 |
| TBIL, M (Q_1_,Q_3_) | 0.90 (0.40,2.10) | 0.80 (0.40,2.00) | 1.00 (0.50,2.70) | Z=1.868 | 0.062 |
| Hematocrit, Mean±SD | 30.40 ± 6.20 | 30.51 ± 6.14 | 30.13 ± 6.35 | t=1.15 | 0.252 |
| RDW, Mean±SD | 16.42 ± 2.76 | 16.16 ± 2.64 | 17.07 ± 2.95 | t=-5.96 | <.001 |

CKD: chronic kidney disease, PaO_2_/FiO_2_: partial arterial oxygen pressure/the fraction of inspired oxygen, SAPS II: the Simplified Acute Physiology Score II, GCS: Glasgow Coma Scale, SOFA: sequential organ failure assessment, SBP: systolic blood pressure, MAP: mean arterial pressure, RBC: red blood count, PLT: platelets, INR: international normalized ratio, WBC: white blood cell count, BUN: blood urea nitrogen, TBIL: total bilirubin, RDW: red cell distribution width
